# Benzoylpaeoniflorin Activates Anti-Inflammatory Mechanisms to Mitigate Sepsis in Cell-Culture and Mouse Sepsis Models

**DOI:** 10.3390/ijms232113130

**Published:** 2022-10-28

**Authors:** Chaeyeong Kim, Hyunchae Sim, Jong-Sup Bae

**Affiliations:** College of Pharmacy, Research Institute of Pharmaceutical Sciences, Kyungpook National University, Daegu 41566, Korea

**Keywords:** benzoylpaeoniflorin, lipopolysaccharide, inflammation, endothelium, CLP, sepsis

## Abstract

*Xuebijing* injection (XBJI) (comprising of five herbs) is a widely used traditional Chinese medicine for sepsis treatment. However, the bioactive components of XBJI and the mechanisms responsible for its sepsis-mitigating action have not been experimentally determined. One of the main bioactive compounds in XBJI—benzoylpaeoniflorin (BPF)—inhibits the expressions of key mediators of inflammation such as nuclear factor kappa B (NF-κB), cyclooxygenase-1 (COX-1), and COX-2. However, its effects on sepsis have not been determined yet. Therefore, here, we investigated the immunomodulatory effect of BPF on severely inflamed endothelial cells, THP-1 macrophages, peritoneal macrophages, and mice. Human umbilical vein endothelial cells (HUVECs) and THP-1-macrophages were activated using lipopolysaccharide (LPS) after pretreatment with BPF. Subsequently, changes in the expression profiles of pro-inflammatory molecules including inducible nitric oxide synthase (iNOS), tumor necrosis factor (TNF)-α, and interleukin (IL)-6 were determined using quantitative real-time polymerase chain reaction (qPCR) and Western blot analysis. Furthermore, we monitored the phosphorylation of NF-kB and mitogen-activated protein kinases (MAPKs) to determine their activation levels. Using the LPS-induced mouse model of sepsis, we studied the effects of BPF on inflammatory cytokine production, pulmonary histopathology, and survival rates. Finally, we evaluated whether BPF protects against cecal ligation and puncture (CLP)-induced sepsis, as it closely mimics human sepsis. BPF pretreatment inhibited LPS-induced increase in mRNA and protein levels of iNOS, TNF-α, and IL-6 in HUVECs and THP-1-macrophages. It also suppressed LPS-mediated phosphorylation of p65, p38, JNK, and ERK. Mice with LPS-induced-sepsis who were treated with BPF had lower serum levels of IL-6, TNF-α, IL-1β, CXCL1, and CXCL2 than the control mice treated with BPF. Histopathology revealed that BPF treatment alleviated LPS-induced lung damage. In addition, in mice given a lethal dose of LPS, BPF treatment showed a dose-dependent improvement in survival rates. BPF treatment dose-dependently inhibited the LPS-induced IL-6, TNF-α, and CXCL1 production in peritoneal macrophages. BPF treatment also dose-dependently improved the survival rates in mice with CLP-induced sepsis. These results show that BPF alleviates LPS-stimulated septic conditions and protects mice from CLP-induced sepsis. Our research marks BPF as a potential drug in the treatment of sepsis and various inflammatory diseases.

## 1. Introduction

Sepsis is defined as life-threatening organ dysfunction caused by a dysregulated host response to infection [1]. Sepsis is a life-threatening condition that occurs when the body’s response to an infection damages its own tissues and the main cause of death in intensive care units. It is characterized by whole-body inflammation, known as “systemic inflammatory response syndrome (SIRS)”. It is increasingly responsible for common illnesses and deaths, especially in older adults, immunocompromised, and severely ill patients [2,3,4]. Pattern recognition receptors (PRRs) are typical innate immune sensors that recognize a variety of pathogen-associated molecular patterns (PAMPs) and damage-associated molecular patterns (DAMPs) [2,3,4]. Overproduction of PAMP and DAMP sensors during sepsis is implicated in multiple organ failure (MOF) [2,3,4]. Toll-like receptor 4 (TLR4) is a PRR that recognizes lipopolysaccharide (LPS) [4]. Upon binding to LPS, TLR4 recruits adaptor proteins such as myeloid differentiation primary response 88 (MyD88) and TIR-domain-containing adapter-inducing interferon-β (TRIF), which activates the inflammatory signaling cascade. This inflammatory signaling cascade further activates nuclear factor kappa B (NF-κB) and mitogen-activated protein kinases (MAPKs), ultimately leading to the expression of inflammatory cytokines, chemokines, and inducible nitric oxide synthase (iNOS) [4]. Although regulated activation of the innate immune response plays a pivotal role in host defense against microbial infection and cellular homeostasis, excessive immune activation can lead to a variety of inflammation-related diseases (such as autoimmune diseases and cytokine release syndrome). Therefore, it is important to medically control the immune overdrive [5]. However, the clinical methods for the treatment of septic injury are limited with no approved drugs currently available for sepsis management. Therefore, finding a safe, non-toxic yet effective treatment is vital to manage sepsis.

**Figure 1:**
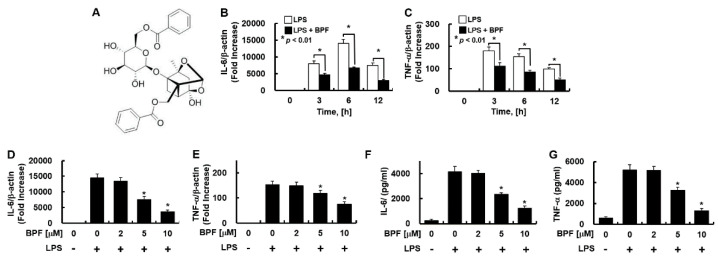
Benzoylpaeoniflorin (BPF) inhibits the expression of inflammatory cytokines in LPS-stimulated HUVECs. (**A**) Structure of BPF; (**B**,**C**) HUVECs were pretreated with BPF (10 μM for 6 h), then LPS (100 ng/mL) was added. After the indicated times, total mRNA was extracted and the expression level of each cytokine and β-actin were determined using real-time PCR; (**D**,**E**) HUVECs were pretreated with the indicated concentrations of BPF for 6 h, then LPS (100 ng/mL) was added. After 12 h, total mRNA was extracted and the expression level of each cytokine and β-actin was determined using real-time PCR; (**F**,**G**) HUVECs were pretreated with BPF at the indicated concentrations for 6 h, then LPS (100 ng/mL) was added. After 24 h, the cell culture supernatant was harvested, and cytokine levels were determined by ELISA. All results are shown as means ± SD of three different experiments with triple samples. * *p* < 0.5 compared to LPS only (**D**–**G**).

Traditional Chinese medicine prescriptions (TCMP) have historically been used to prevent and treat various diseases. *Xuebijing* injection (XBJI) is a clinical TCMP. XBJI is an aqueous extract of five traditional medicinal herbs—viz. *Flos Carthami, Radix Paeoniae Rubra, Radix Salviae Miltiorrhizae, Rhizoma Chuanxiong,* and *Radix Angelicae Sinensis* [6,7]. XBJI was approved by China Food and Drug Administration (CFDA) in 2004 for the clinical treatment of SIRS, MOF, and sepsis [8]. It is effective in suppressing the cytokine storm, reducing inflammation, activating blood circulation, detoxifying, and reducing organ damage [9]. The four active compounds of XBJI are safflor yellow A, oxypaeoniflorin, benzoylpaeoniflorin (BPF, Figure 1A), and ferulic acid. Among them, BPF inhibits NF-κB activity and cyclooxygenase-1 (COX-1) and COX-2 expressions [10,11] and it has anti-anaphylactic functions [12]. However, the in vivo immunomodulatory and immunoregulatory effects and mechanisms of BPF alone have not been studied yet. Therefore, here, we tried to determine the immunomodulatory effects of BPF and its molecular mechanisms in endothelial cell culture, THP-1-macrophages, peritoneal macrophages, and murine sepsis models.

## 2. Results

### 2.1. BPF Is Non-Cytotoxic and Reduces LPS-Induced Inflammatory Cytokine Production by HUVECs and THP-1-Macrophages

Endothelial cells elicit a strong immunological response and secrete a variety of inflammatory cytokines in response to LPS stimulation [13]. To investigate whether BPF regulates this inflammatory response, HUVECs pretreated with BPF were stimulated with LPS. The IL-6 and TNF-α expression levels were measured. BPF pretreatment significantly suppressed the mRNA expression of IL-6 and TNF-α by LPS in a time- (Figure 1B,C) and dose-dependent (Figure 1D,F) manner. BPF pretreatment also reduced the LPS-induced increase in the IL-6 and TNF-α protein levels in a dose-dependent manner (Figure 1F,G).

**Figure 2:**
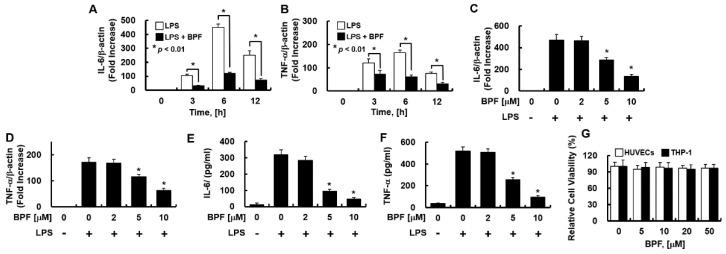
BPF inhibits the expression of inflammatory cytokines in LPS-stimulated THP-1-macrophages. (**A**,**B**) THP-1-macrophages were pretreated with BPF (10 μM, for 6 h), then LPS (100 ng/mL) was added. After the indicated times, total mRNA was extracted and the expression level of each cytokine and β-actin were determined using real-time PCR; (**C**,**D**) THP-1-macrophages were pretreated with the indicated concentrations of BPF for 6 h, then LPS (100 ng/mL) was added. After 12 h, the total mRNA was extracted and the expression level of each cytokine and β-actin was determined using real-time PCR; (**E**,**F**) THP-1-macrophages were pretreated with BPF at the indicated concentrations for 6 h, then LPS (100 ng/mL) was added. After 24 h, the cell culture supernatant was harvested, and cytokine levels were determined using ELISA. (**G**) HUVECs or THP-1-macrophages were treated with the indicated concentrations of BPF for 48 h. Cell viability was determined using the MTT assay. All results are shown as means ± SD of three different experiments with triple samples. * *p* < 0.5 compared to LPS only (**D**–**F**).

Consistently, in THP-1-macrophages, BPF pretreatment significantly inhibited LPS-induced IL-6 and TNF-α mRNA expression in a time- (Figure 2A,B) and dose-dependent manner (Figure 2C,D), and effectively suppressed the LPS-induced IL-6 and TNF-α production (Figure 2E,F). Moreover, the MTT assay revealed that 48 h exposure to BPF (<50 μM) was non-cytotoxic to both cell types (Figure 2G).

### 2.2. BPF Inhibits LPS-Induced Expression of Inducible Nitric Oxide Synthase (iNOS) and LPS + IFN-γ-Induced Production of Nitric Oxide (NO) by HUVECs

**Figure 3:**
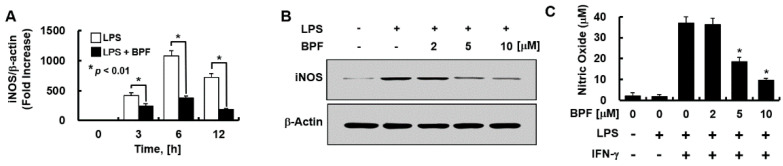
BPF inhibits the expression of iNOS and the production of nitric oxide in HUVECs. (**A**) HUVECs were pretreated with BPF (10 μM for 6 h), then LPS (100 ng/mL) was added. After the indicated times, total mRNA was extracted and the expression level of iNOS and β-actin was determined using real-time PCR; (**B**) HUVECs were pretreated with the indicated concentrations of BPF for 6 h, then LPS (100 ng/mL) was added. After 24 h, the cellular protein was extracted and iNOS and β-actin levels were determined using Western blots; (**C**) HUVECs were pretreated with the indicated concentrations of BPF for 6 h, then LPS (100 ng/mL) and IFN-γ (200 ng/mL) were added under the indicated conditions. After 24 h, the cell culture supernatant was harvested, and nitric oxide levels were determined using the Griess reaction assay (**A**,**C**). All results are shown as means ± SD of three different experiments with triple samples. * *p* < 0.5 compared to LPS + IFN-γ (**C**); (**B**) The results are from one representative experiment of three independent experiments.

We evaluated whether BPF regulates the expressions of iNOS (mainly responsible for NO synthesis in the endothelium) and NO. BPF pretreatment significantly inhibited LPS-induced iNOS mRNA levels in a time- (Figure 3A) and iNOS protein levels in a dose- (Figure 3B) dependent manner in HUVECs. We found that LPS stimulation was not enough for NO production by HUVECs; interferon-γ (IFN-γ) had to be co-applied with LPS to stimulate NO production. BPF markedly inhibited LPS + IFN-γ-induced NO production in a dose-dependent manner in HUVECs (Figure 3C), consistent with the iNOS expression results.

### 2.3. BPF Reduces LPS-Induced Phosphorylation of NF-κB (p65) and MAPK (p38, JNK, and ERK), in HUVECS and THP-1-Macrophages

**Figure 4:**
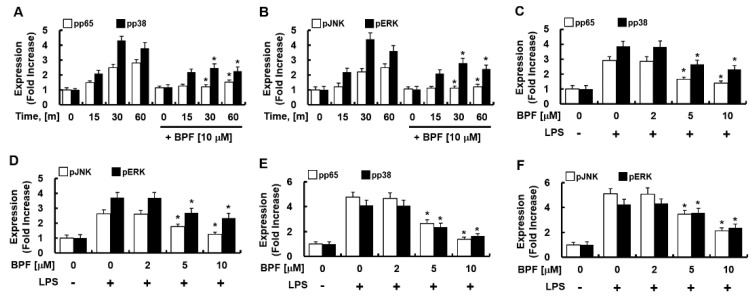
BPF inhibits the phosphorylation of NF-κB and MAP kinase in LPS-stimulated macrophages. (**A**,**B**) HUVECs were pretreated with BPF (10 μM for 6 h), then LPS (100 ng/mL) was added. Cellular protein was extracted at the indicated times; (**C**,**D**) HUVECs were pretreated with the indicated concentrations of BPF for 6 h, then LPS (100 ng/mL) was added. After 1 h, the cellular protein was extracted. (**E**,**F**) THP-1-macrophages cells were pretreated with the indicated concentrations of BPF for 6 h, then LPS (100 ng/mL) was added. After 1 h, the cellular protein was extracted (**A**–**C**) The levels of the indicated proteins were determined using ELISA. All results are shown as the means ± SD of three different experiments with triple samples. * *p* < 0.5 compared to LPS only.

Since MAPK-activated transcription factor NF-kB and activator protein 1 (AP-1) mediate the expression of IL-6, TNF-α, and iNOS during PRR activation, we next determined the effect of BPF on the phosphorylation of NF-κB (p65) and MAPKs (such as p38, JNK, ERK) in LPS-stimulated HUVECs and THP-1-macrophages. BPF pretreatment considerably reduced the LPS-induced activation of p65, p38, JNK, and ERK (Figure 4A–D) in HUVECs and in THP-1-macrophages (Figure 4E,F) in time- and dose-dependent manner. Thus, BPF suppresses LPS-induced inflammation by inhibiting activation of inflammation-related transcription factors in human cells.

### 2.4. BPF Reduces Cytokine Production, Pulmonary Damage, and Mortality in LPS-Induced Mouse Septic Shock Model

**Figure 5:**
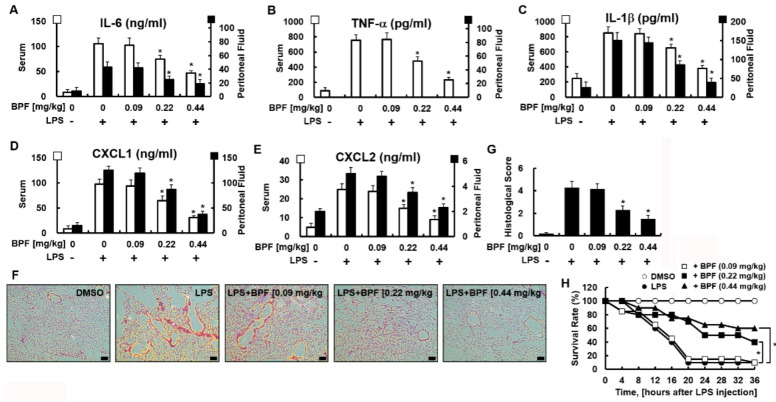
BPF alleviates excessive cytokine production and tissue damage of the lungs and improves the survival rate in the LPS-induced mouse septic shock model. (**A**–**E**) Mice were injected with 2.5 mg/kg of LPS (intraperitoneal injection) and DMSO or BPF (0.09, 0.22 or 0.44 mg/kg, intravenous injection). After 4 h, the mice were euthanized. Blood and peritoneal fluid were collected and the levels of the indicated inflammatory cytokines were determined using ELISA (n = 5). (**F**,**G**) Mice were injected with 2.5 mg/kg LPS (intraperitoneal injection) and DMSO or BPF (0.09, 0.22 or 0.44 mg/kg, intravenous injection). After 24 h, the mice were euthanized and the left lung lobes were collected and fixed in formalin, followed by staining with H&E. H&E staining of lung tissues from each group was conducted, and representative images from three independent experiments conducted on three different days are shown. The bar represents 200 μm. Histopathological scores were obtained using an arbitrary scoring index based on the degree of inflammatory cell infiltration and the extent of the lesion area (n = 5). (**H**) Mice were injected with 25 mg/kg of LPS (intraperitoneal injection) and DMSO or BPF (0.09, 0.22 or 0.44 mg/kg, intravenous injection). The survival of the mice was monitored every 4 h for 36 h and the survival rates were expressed as a percentage (n = 20). All results are shown as the means ± SD of three different experiments with triple samples. * *p* < 0.5 compared to LPS only.

We next verified whether these in vitro results could be replicated in vivo. We used the LPS-induced mouse septic shock model. We found that BPF protected against LPS-induced sepsis. BPF (*i.v.* 0.22 or 0.44 mg/kg) treatment significantly reduced the cytokine levels in serum and peritoneal fluid (Figure 5A–E) and lung tissue damage (Figure 5F,G) in LPS-stimulated mice (LPS sublethal dose, 2.5 mg/kg). Furthermore, BPF protected against a lethal dose of LPS. The effect of BPF treatment on mice injected with LPS (lethal 25 mg/kg) was studied by monitoring every 4 h for 36 h. While only two out of the 20 control (lethal LPF + no BPF treatment) mice survived, eight out of 20 and 12 out of 20 mice administered lethal LPS + 0.22 mg/kg BPF and lethal LPS + 0.44 mg/kg BPF survived, respectively (Figure 5H). Therefore, the administration of BPF significantly reduced LPS-induced mortality in mice.

### 2.5. BPF Inhibits the CLP-Induced Cytokines Production by Peritoneal Macrophages and Improves Survival Rate of Mice with CLP-Induced Sepsis

**Figure 6:**
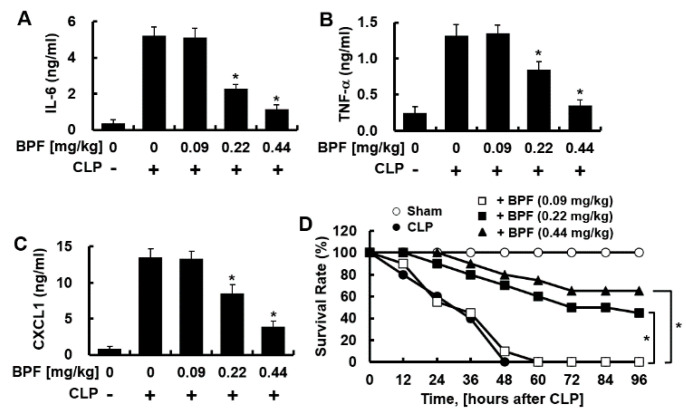
BPF suppresses CLP-induced cytokine production of peritoneal macrophages and improves the mortality of CLP mice. (**A**–**C**) CLP-operated mice were injected with DMSO or BPF (0.09, 0.22, or 0.44 mg/kg, intravenous injection). After 12 h, the mice were euthanized. Peritoneal macrophages were collected from CLP-operated mice and the levels of the indicated inflammatory cytokines were determined using ELISA (n = 5). All results are shown as means ± SD of three different experiments with triple samples. * *p* < 0.1 compared to CLP only; (**D**) CLP surgery was conducted on mice and the indicated reagents were administered every 24 h (n = 20). The survival of mice was monitored for 96 h. * *p* < 0.5.

Sepsis peritoneal macrophages modulate SIRS by producing anti-inflammatory cytokines [14]. The CLP-induced sepsis model is the golden standard for the animal sepsis model mimicking human sepsis [15]. Therefore, we checked whether BPF inhibits the CLP-induced production of inflammatory cytokines by peritoneal macrophages. BPF treatment indeed inhibited the CLP-induced production of IL-6, TNF-α, and CXCL1 by sepsis-induced peritoneal macrophages (Figure 6A–C) (consistent with the results in HUVECs and THP-1-macrophages). To determine whether BPF affected the survival of mice with CLP-induced sepsis, we administered BPF intravenously to the CLP-operated mice and monitored their survival for 96 h. While all of the control mice (treated with DMSO) died 48 h after CLP surgery, nine out of 20 BPF (0.22 mg/kg)-injected mice and 13 out of 20 BPF (0.44 mg/kg)-injected mice survived for 96 h post CLP surgery (Figure 6D).

## 3. Discussion

Over the years, many traditional Chinese herbal medicines have been adopted for medical use due to their verified clinical effects; these have been widely studied and have well-known safety profiles with low toxicity. Natural chemicals extracted from these medicinal herbal plants have been recognized as pharmacologic supplements in various inflammatory disorders [16,17]. These chemicals inhibit inflammatory mediators (NF-κB, MAP Kinases), immune-related transcription factors, and expressions of inflammatory cytokines (TNF-α or IL-series) [18,19]. XBJI treatment reduces mortality, anal temperature, and the expression of inflammatory factors such as TNF-α, IL-1β, and IL-6 when administered to mouse sepsis models [8,9]. Since NF-κB inhibition is the key action of XBJI in alleviating sepsis, six ingredients with NF-κB inhibitory properties—senkyunolide I, paeoniflorin, danshensu, safflor yellow A, oxypaeoniflorin, and BPF—were identified in XBJI using UPLC-Q/TOF based protocols [10]. BPF is a benzoylated monoterpene glycoside. It was originally reported from Chinese Paeony root [20]. However, it was also reported from Radix Paeonia Rubra, which is the dried root of *Paeonia lactiflora* Pallas [21]. BPF in XBJI is speculated to be derived from Radix Paeoniae Rubra, one of the medicinal herbs in XBJI. In particular, we focused on BPF as a sepsis drug candidate because among the six NF-κB inhibitors in XBJI, BPF is (1) the most abundant component, (2) the most effective inhibitor of NF-κB activity, and (3) an inhibitor of COX-1 and COX-2 activities. Thus, we aimed to clarify novel in vitro and in vivo sepsis-mitigating and anti-inflammatory effects of BPF. Here, we show that BPF suppresses the LPS-induced inflammatory cytokine release by HUVECs and TPH-1-macrophages. It also exerts anti-inflammatory effects on LPS- and CLP-induced sepsis. Thus, it protects mice from sepsis. 

Long-term dysregulation of inflammatory genes including inflammatory cytokines, iNOS, and COX leads to chronic inflammation, which contributes to a variety of inflammatory diseases [22]. Furthermore, MAPKs (such as p38, JNK, and ERK) respond to various stimuli by inducing the expression of pro-inflammatory mediators. The amplified inflammatory responses can thus be seen as an interplay of the NF-κB and MAPKs pathways. Logically, drugs for inflammatory diseases must target MAPKs activities [22]. Thus, agents that modulate anti-inflammatory mediators have emerged as good candidates for developing therapeutics for a variety of inflammatory diseases. We found, here, that BPF exerts several anti-inflammatory effects by suppressing LPS-induced: (1) iNOS and NO generation; (2) TNF-α and IL-6 expressions; and (3) JNK, ERK, and p38 MAPK activation. BPF also suppresses the PRR activation-induced strong expression of COX-1 and -2 [11]. Overall, BPF suppresses the increase in iNOS and COX-2 levels by inhibiting the LPS-stimulated activation of JNK and p38 MAPK. Thus, BPF protects against the effects of the inflammatory immune response via blocking of the NF-κB-JNK/P38K pathway, which in turn inhibits the expression of pro-inflammatory genes.

The severity and prognosis of inflammatory diseases are closely associated with exaggerated inflammatory responses [23]. In particular, sepsis develops when appropriate host responses that are initially amplified to tackle an infection are combined with poor control of subsequent infections, leading to an imbalance between inflammatory and anti-inflammatory responses [23,24]. The expression and regulation of inflammatory and anti-inflammatory cytokines are significantly related to the prognosis of patients with sepsis [24]. For example, the excessive production of pro-inflammatory cytokines launched by the host for effective immune response can lead to cytokine storm and subsequent septic shock, MOF, and death [24]. This kicks in the overproduction of anti-inflammatory cytokines (such as IL-10 and IL-1 receptor antagonists) to inhibit excessive inflammatory responses and maintain host homeostasis, which leads to the inhibition of the immune system, thus promoting bacterial burden and increasing host mortality [23,24]. Therefore, treatment of patients with sepsis entails mitigating the overactivation of pro- and anti-inflammatory responses to alleviate septic shock and bacterial burden. 

Based on the results of the anti-inflammatory effects of BPF on THP-1-macrophages, we hypothesized that BPF would effectively inhibit the release of pro-inflammatory cytokines in sepsis. We verified this using the LPS-induced mouse septic shock model. As expected, BPF significantly suppressed LPS-induced IL-6, TNF-α, CXCL1, and CXCL2 production and mortality in the mice. Moreover, our data showed that BPF significantly inhibits the LPS-induced production of inflammatory cytokines by peritoneal macrophages, which play an important role in SIRS [25]. Even in the CLP-induced mouse sepsis model, the administration of BPF inhibited the production of pro-inflammatory cytokines by peritoneal macrophages and improved the survival rate. 

Xigris, used to treat sepsis, was withdrawn from market in 2011 due to issues with side effects and the lack of efficacy [26] (licensed by Food and Drug Administration (FDA) in 2001 and by European Medical Agency (EMA) in 2002) [27]. Since then, there has been no approved treatment for sepsis. Therefore, the development of alternative drugs for sepsis has become imperative. Although studies on the side effects and toxicity of BPF are lacking (and need to be conducted in future), the results of our study indicate that BPF will be a good candidate for the treatment of sepsis and various inflammatory diseases.

In conclusion, BPF is protective as it significantly inhibits LPS-induced inflammatory cytokine production in endothelial cells, ameliorates lung tissue damage, and reduces mortality in septic mice. Therefore, BPF may be a potential drug in treating various vasculitis-related diseases.

## 4. Materials and Methods

### 4.1. Reagents and Antibodies

BPF (purity > 98%) was purchased from Selleck Chemicals (Houston, TX, USA). LPS derived from bacteria (serotype: 0111:B4, L5293) and phorbol 12-myristate 13-acetate (PMA) were purchased from Sigma-Aldrich (St. Louis, MO, USA). Anti-iNOS and anti-β-actin were obtained from Cell Signaling Technology (Beverly, MA, USA).

### 4.2. Cell Culture

Primary human umbilical vein endothelial cells (HUVECs) were obtained from Cambrex Bio Science (Charles City, IA, USA) and maintained as previously described [28]. Cells were cultured in EBM-2 basal media (Cambrex Bio Science) at 37 °C under 5% CO_2_. THP-1 cells, derived from a human monocyte line, were purchased from the ATCC (Manassas, VA, USA) and cultured as previously described [29]. Differentiation of THP-1 cells into macrophages was induced by incubating them in complete medium containing PMA (50 μM) for 48 h followed by incubation in complete medium without PMA for 48 h.

### 4.3. Animal Experiments

Male C57BL/6 mice (8-week-old, 27 ± 0.5 g) were purchased from Orient Bio Co. (Sungnam, Republic of Korea). All animal experiment protocols were approved by the Animal Care Committee at Kyungpook National University prior to conducting the study (IRB No. KNU 2020-107). Each animal sepsis model was established as below:

*LPS-induced septic shock model*: LPS (2.5 mg/kg) was administered to mice by intraperitoneal injection. Simultaneously, BPF was administered intravenously. For cytokine analysis, the mice were euthanized 4 h later. The blood and ascitic fluid were collected and centrifuged at 9500× *g* at 4 °C for 10 min. The supernatant was used for cytokine analysis using ELISA. For lung histological analysis, the septic shock was extended to 24 h.

*LPS-induced lethal septic shock model*: A lethal dose of LPS (25 mg/kg) was administered to mice by intraperitoneal injection. Simultaneously, BPF was administered intravenously. Mouse survival was observed every 4 h for 36 h.

*Cecal Ligation and Puncture (CLP) sepsis model*: Mice were prepared as described previously [30]. Sham operated animals underwent laparotomy without ligation and puncture of the cecum. BPF was injected intravenously and the mice were observed every 12 h for 96 h.

### 4.4. Murine Peritoneal Macrophage Isolation

Mice were administered 3 mL of 3% thioglycolate by intraperitoneal injection. Three days later, the mice were euthanized. Peritoneal macrophages were collected and washed with PBS. Subsequently, they were seeded in 48-well plates at a density of 5 × 10^5^ cells/mL.

### 4.5. Methyl-Thiazolyl-Tetrazolium (MTT) Cell Viability Assay

The cultured HUVECs and THP-1-macrophages were incubated with BPF at the indicated concentrations in serum-free medium. After 48 h, the 3-(4,5-dimethylthiazol-2-yl)-2,5-diphenyltetrazolium bromide (MTT) assay was performed to estimate the cell viability [31,32].

### 4.6. Enzyme-Linked Immunosorbent Assay (ELISA)

The activities of the pp65, pp38, pJNK, and pERK cellular proteins were determined by using commercially available ELISA kits (Cell Signaling Technology). The levels of IL-6, TNF-α, CXCL1, and CXCL2 in the cell culture supernatants were determined by using ELISA kits (R&D System, Minneapolis, MN, USA) according to the manufacturer’s instructions.

### 4.7. Quantitative Real-Time Polymerase Chain Reaction (qPCR)

RNA was purified using TRIzol Reagent (Thermo Fisher Scientific, Carlsbad, CA, USA) extraction. The purified RNA was reverse transcribed with a PX2 Thermal Cycler (Thermo Fisher Scientific) using 0.5 mg/mL of the oligo (dT)-adapter primer (Thermo Fisher Scientific) and M-MLV reverse transcriptase (Thermo Fisher Scientific) in a 20 μL reaction mixture. The primer sequences were as follows: IL-6; forward, 5′-GCA CTG GCA GAA AAC AAC CT-3′ and reverse, 5′-TCA AAC TCC AAA AGA CCA GTG A-3′. TNF-α; forward, 5′-CCC AGG GAC CTC TCT CTA ATC-3′ and reverse, 5′-ATG GGC TAC AGG CTT GTC ACT-3′. iNOS; 5′-TCC AGG AGG ACA TGC AGC AC3′ and reverse, 5′-CGC CCT TCC GCA GTT CT-3′. β-actin; forward, 5′-CCT GGC ACC CAG CAC AAT-3′ and reverse, 5′-GCC GAT CCA CAC GGA GTA CT-3′. Quantitative gene expression levels were normalized to the expression levels of β-actin.

### 4.8. Immunoblotting

Lysis buffer containing Nonidet P-40, complete protease inhibitor cocktail (Roche, Mannheim, Germany), 2 mM dithiothreitol, and phosphatase inhibitor cocktail 2 (Sigma-Aldrich) was used to lyse the cells. The proteins in the treated cell lysate were separated using SDS-PAGE and transferred to nitrocellulose membranes. Transferred proteins were incubated with primary antibodies corresponding to each target protein and then with the relevant secondary antibodies.

### 4.9. Measurement of Nitrite Levels in Cell Culture Supernatants

The conditioned cell culture supernatants were harvested, and nitric oxide concentrations were determined using the Griess reaction assay.

### 4.10. Hematoxylin and Eosin (H&E) Staining

Five mice were used for histopathological analysis. Mice from the LPS-induced septic shock model were euthanized and the left lobes of lungs were fixed in 10% neutral formalin for 24 h, followed by tissue processing and paraffin embedding. The paraffin blocks were sectioned into 2-μm thick sections and stained with H&E. Histopathological scoring was conducted by three experimental veterinary medicine experts using a random score index based on the degree of inflammatory cell infiltration and the extent of the injury area (0, normal; 1, mild; 3, moderate; 5, severe) with slight modifications from previous studies [33,34].

### 4.11. Statistical Analysis

Results are expressed as the mean ± standard deviation (SD) of three independent experiments. In order to assess the data distribution, the normality test (Shapiro–Wilk test) was performed. The statistical test was selected based on the results of the normality test. Results were analyzed using ANOVA (one-way) followed by Tukey’s post hoc tests and differences at *p* < 0.05 were considered significant. The Kaplan–Meier method was used to compare differences in the survival outcomes following LPS- or CLP-induced sepsis experiments.

## Data Availability

The data presented in this study are available upon reasonable request from the corresponding author.
